# Effect of ozone therapy on post-operative pain following mandibular third molar surgical extraction: a split-mouth randomized clinical trial

**DOI:** 10.1007/s00784-025-06681-y

**Published:** 2025-12-09

**Authors:** Luca Guaschino, Alessandro Vanzanelli, Piermarco Babando, Andrea Ricotti, Guglielmo Amedeo Ramieri, Andrea Roccuzzo, Paolo Appendino

**Affiliations:** 1 Department of Dentistry and Oral Surgery, Mauriziano Umberto I Hospital, Turin, Italy; 2 Clinical Trial Unit, Mauriziano Umberto I Hospital, Turin, Italy; 3 Division of Maxillofacial Surgery, University of Torino, Torino, Italy; 4 Perio-Implant Innovation Center, Institute for Integrated Oral, Craniofacial and Sensory Research - National Clinical Research Center of Stomatology, Ninth People’s Hospital, Shanghai Jiao Tong University School of Medicine, 4F Building 1, 115 Jinzun Road, Pudong Research Campus, Shanghai, 200115 China; 5 College of Stomatology, National Center of Stomatology, National Clinical Research Center for Oral Diseases, Shanghai Key Laboratory of Stomatology, Shanghai Jiao Tong University, Shanghai, China; 6 Department of Periodontology, School of Dental Medicine, University of Bern, Bern, Switzerland

**Keywords:** Clinical trial, Mandibular third molar, Oral surgery, Ozone therapy, Pain, Quality of life, Third molar extraction, Trisma

## Abstract

**Objectives:**

To evaluate the efficacy of ozone therapy on pain reduction, trisma and number of analgesic tablets taken following surgical removal of impacted mandibular third molar.

**Methods:**

A split-mouth randomized clinical trial was conducted in 54 patients in need of bilateral impacted mandibular third molars (Pell-Gregory class II-B) extraction. Test sites were treated with adjunctive ozone therapy to the surgical extraction, while controls underwent surgical intervention only (time between surgeries: 3 weeks). Study primary outcome measure was the difference in perceived pain recorded using a Numerical Rating Scale (NRS) at 7 days after surgery. In addition, functional limitation in mouth opening (mm), number of analgesic tablets taken within one week after surgery as well as the patient’s perceived quality of life (Oral Health Impact Profile (OHIP-14) were evaluated.

**Results:**

Fifty patients (20 males and 30 females; median age: 21 years; all non-smokers) completed the study. Mean reported perceived pain decrease from 5.06 to 0.58 (test group) and from 5.62 to 1.08 (control group) between day 1 and 7. No statistically significant differences were detected between the two groups at any time points (*p* > 0.05). At day 7, mean mouth opening value was 40.8 vs. 36.6 mm in test and control group respectively (mean difference: 4.24 mm; *p* < 0.001; 95% CI: 6.55–1.93). The same trend was detected for the of number of analgesic tablets taken (median: 4 vs. 7; *p* = 0.01). Finally, OHIP-14 values did not differ between groups (*p* > 0.05).

**Conclusion:**

Ozone therapy had a beneficial clinical effect in terms of mouth opening and in the number of analgesic tablets intake at 7 days after surgical removal of impacted mandibular third molar, despite the lack of adjunctive benefit on patients perceived pain reduction.

**Clinical relevance:**

The clinical use of ozone therapy as adjunct to surgical removal of impacted mandibular third molar might be considered in light of the promising positive reported results in terms of mouth opening reduction and analgesic intake.

**Clinical trial registration number:**

NCT05949476

## Introduction

Mandibular third molar extraction is one of the most common intervention in oral and maxillofacial surgery and is associated with several post-operative sequelae [13]; Parrini, De Ambrosi, & Chisci [21]),. Several reasons have been advocated to bony impaction of the lower third molars: insufficiency of the retromolar space, malposition of the tooth germ, hereditary factors, lack of sufficient eruption force, as well as the phylogenetic regression of the dimensions of the jaw [3, 17]. When focusing on the most common reasons for tooth removal, they include caries, pericoronitis, periodontal problems, and the formation of odontogenic cysts (Chisci, Parrini, Baldini, & Chisci [6],; Gloria, Douglas-de-Oliveira, LDA, Falci, & Dos Santos [12]),. The most common complications that occur following the extraction of impacted lower third molars are post-operative pain, edema, trisma, alveolitis, and infection of the post-extraction site, as well as a worsening of the quality of life perceived in the days following the extraction [10] (Cho, Lynham, & Hsu [7]), (Slade, Foy, Shugars, Phillips, & White [28]),. To manage post-operative discomfort, various strategies have been developed to minimize clinical manifestations after surgery both through surgical methods, instrumental methods and through a pharmacological approach by inhibiting the synthesis and/or release of inflammatory mediators of acute inflammation: these are corticosteroids and non-steroidal anti-inflammatory drugs (FANSs)(de Barros et al., [9]; Giansiracusa, Parrini, Baldini, Bartali, & Chisci [11]),. However, the use of corticosteroids or NSAIDs has been associated with some adverse effects [10] [5]; Kazancioglu, Kurklu, & Ezirganli [15],; Slade et al., [28]. Consequently, it is of clinical interest to identify protocols complementary to pharmacological therapy, like ozone therapy which is treatment based on a mixture of oxygen and ozone as a therapeutic agent. Ozone (O3) is a gas composed of three oxygen atoms, whose molecular weight is 47.98 g/mol. It is a dynamically unstable substance due to the presence of mesomeric states, which rapidly liberate single oxygen atoms [4, 16]; Nogales, Ferrari, Kantorovich, & Lage-Marques [19],. Clinically, ozone can be applied in three forms: gaseous, aqueous, and oily (gel). The biological effects of ozone include improving oxygen metabolism, increasing cellular energy and immunomodulatory properties, and enhancing the antioxidant defense system [2]. Consequently, considering the aforementioned characteristics, its application in oral surgery has been investigated: Kazancioglu et al. evaluated patients for post-operative trisma and swelling following third molar surgery. As a conclusion of that study, they reported that no differences were found between the two sides for mouth opening or swelling [15]. The degree of pain and the number of analgesic tablets taken was significantly lower for the study side. Ramos Gloria et al. using ozonized water during third molar surgery, had satisfactory effects on management of pain, edema and trisma after surgical removal of the third molar [12]. Based on these studies, ozone therapy appears to be effective in reducing post-operative related discomfort in third molar surgery. However, based on the conclusions of a recent systematic review highlighting the small number of studies in the literature [5], the aim of this split-mouth randomized clinical trial was to determine the efficacy of ozone therapy on perceived pain reduction, trismus and number of analgesic tablets taken following surgical removal of impacted mandibular third molar.

## Materials and methods

The study protocol was submitted to and approved by the Institutional Ethics Committee (N. 249/2022). The investigation was conducted according to the revised principles of the Helsinki Declaration (2018), and signed informed consent was obtained from each patient before entering the study. The trial was registered on ClinicalTrials.gov (NCT05949476).

### Study design, group allocation and randomization

This study was designed as a single-center split-mouth randomized clinical trial. It was performed at the Department of Dentistry and Oral Surgery, Mauriziano Umberto I Hospital, Turin, Italy, as part of the routine daily workflow. Subjects potentially available for enrollment were informed in advance with dedicated information sheets on intervention details and on data protection. Those who agreed to participate in the study completed the personalized informed consent form and were provided with the data collection form to be completed in the days following surgery.

**Fig. 1:**
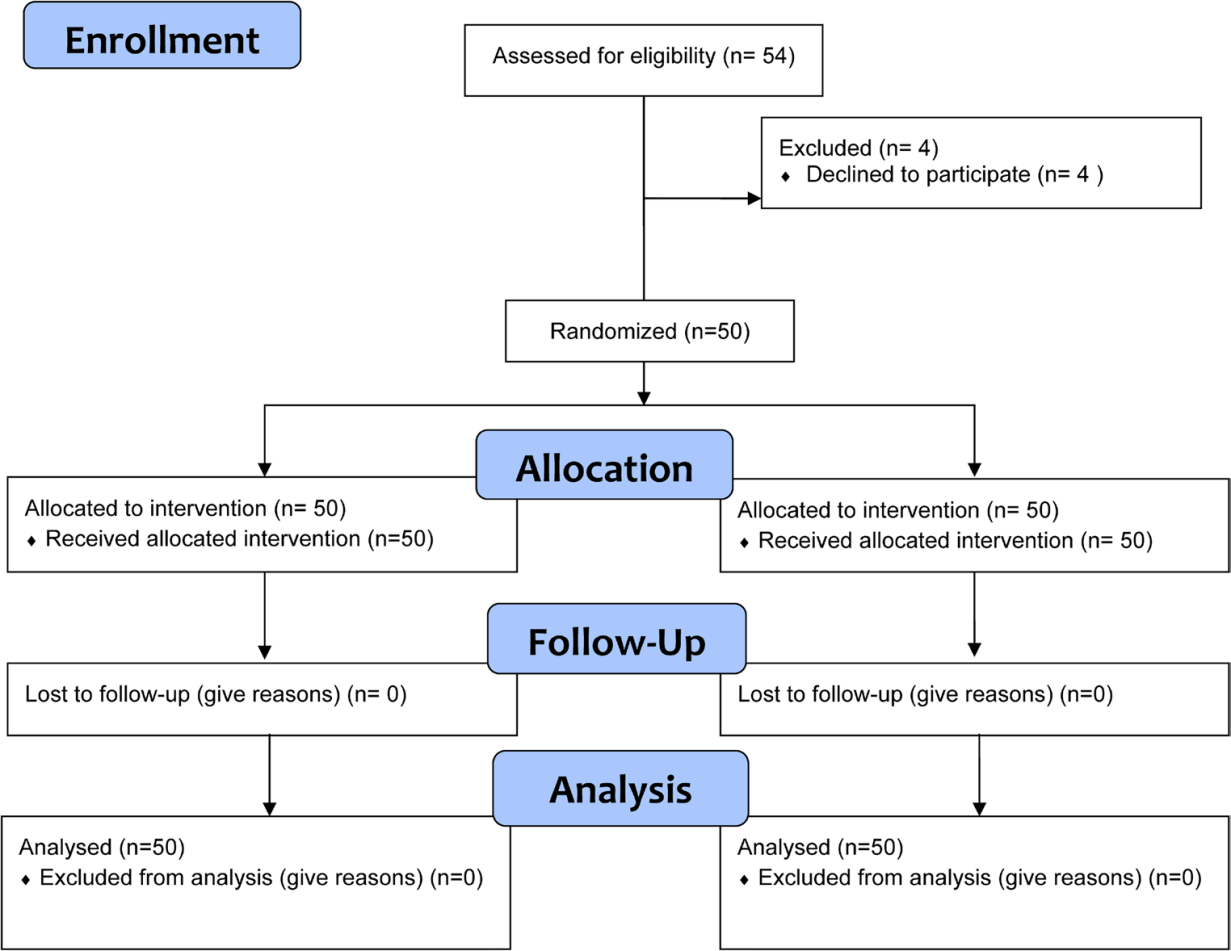
Study flow-chart

The present study was designed as a prospective, split-mouth, randomized, controlled, clinical trial with a parallel design (1:1 ratio). The study flow chart is reported in Fig. 1. Data reporting followed the Consolidated Standards of Reporting (CONSORT) guidelines. Patients’ sites were randomly allocated to the test (i.e., ozone therapy) or control (i.e., no treatment) group by toss coin by an external investigator not involved in the surgical intervention or in the outcome evaluations prior performing local anesthesia. Sites randomized to the test group were operated first. The time interval between the first and second surgery was 3 weeks [24]; Siddiqi, Morkel, & Zafar [26]),.

### Study population

Subjects referred to the Department of Dentistry and Oral Surgery, Mauriziano Umberto I Hospital, Turin, Italy were consecutively screened for recruitment between July 2022 and July 2024. One experienced investigator (P.A.) evaluated all subjects and was responsible for patients’ enrollment following the assessment of the inclusion and exclusion criteria.

### Inclusion criteria

- Males and female subjects aged between 18 and 30 years.
- Need of surgical removal of both impacted lower third molars according to Pell-Gregory class II-B [22] for orthodontic purposes.
- Absence of systemic diseases.

### Exclusion criteria

- Presence of any systemic disease or condition which might interfere with post-operative healing.
- Pregnancy and breastfeeding.
- Ozone allergy.

### Surgical intervention

Prior to surgery, for extra-oral antisepsis, a skin solution containing chlorhexidine gluconate and 96% ethyl alcohol (alcoholic Neoxinal 0.5% + 70%) was applied. Local anesthesia was performed with the regional inferior and lingual alveolar nerve block technique, with a complement of vestibular nerve anesthesia. A maximum volume of up to 5.4 mL of a local anesthetic solution containing Mepivacaine hydrochloride 20 mg/mL and 1:100,000 adrenaline (equivalent to 3 vials of 1.8 mL local anesthetic solution) was used for each intervention.

All surgical interventions were performed following a standardized surgical protocol by two experienced oral surgeons (L.G, A.V.). More specifically, following creation of an envelope flap with a distal releasing incision and elevation of a full-thickness flap, osteotomy with a Lindeman-type drill mounted on a straight handpiece under constant irrigation with physiological solution was performed. Tooth extraction was carried out with the aid of Bein-type levers followed by an accurate curettage, bone regularization and cleaning of the surgical area through abundant irrigation with physiological solution. Finaly, a resorbable suture (Polyglactin 910–4.0) was used [30].

**Fig. 2:**
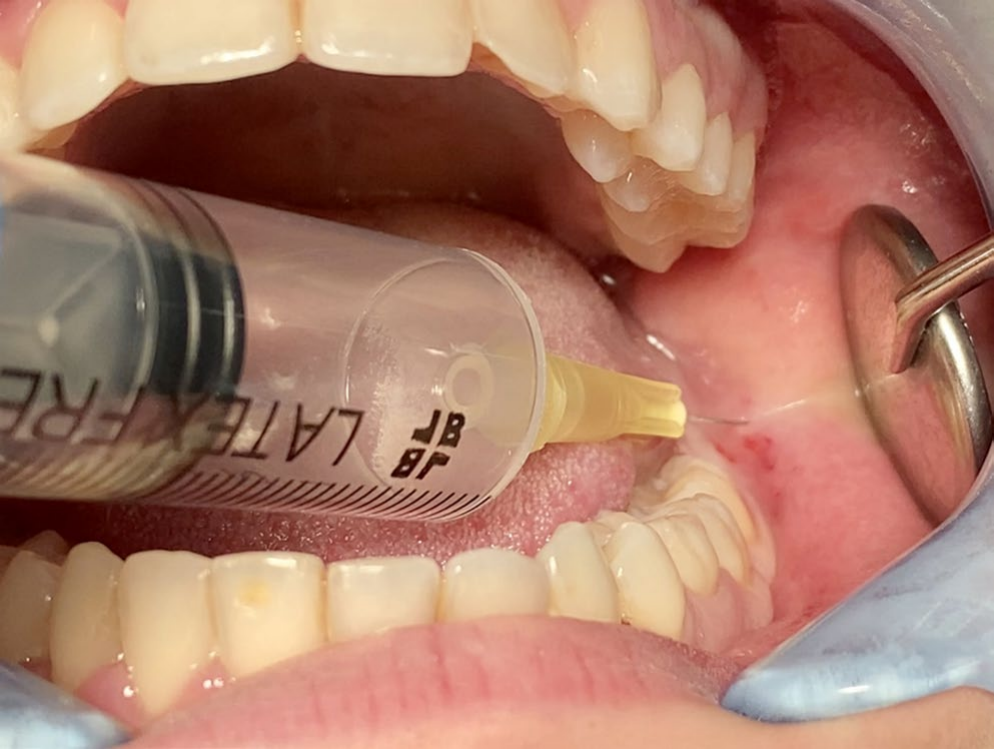
Injection of gaseous ozone (5 μg/ml) after anesthesia around the incision area

**Fig. 3:**
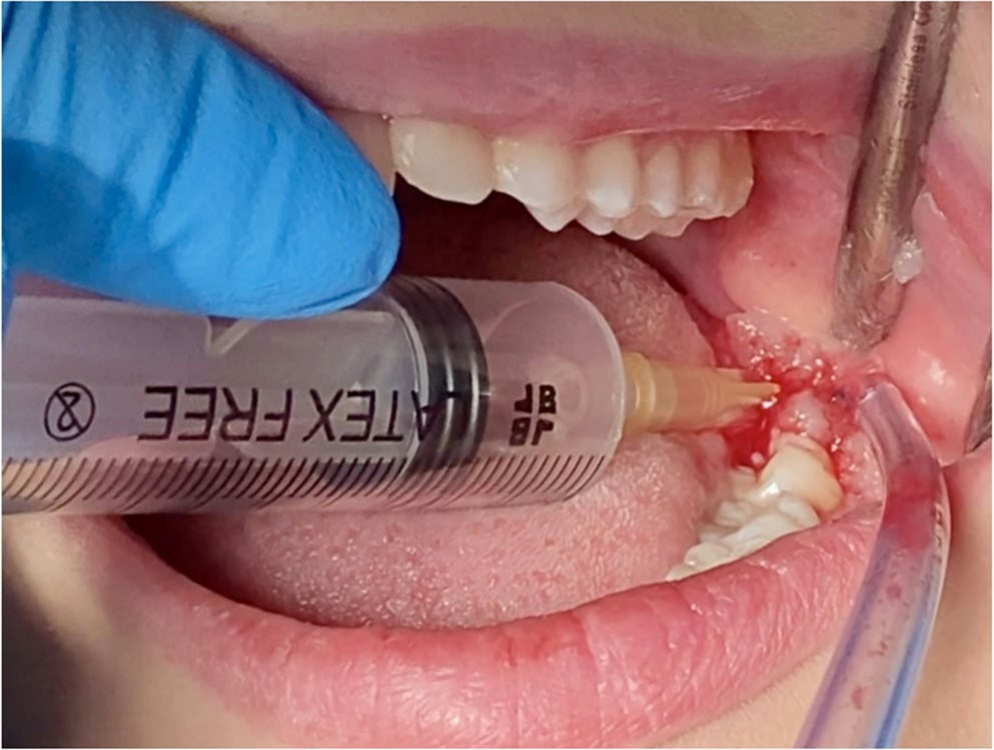
Ozone injection in the post-extraction socket (40 μg/ml) after suturing

**Fig. 4:**
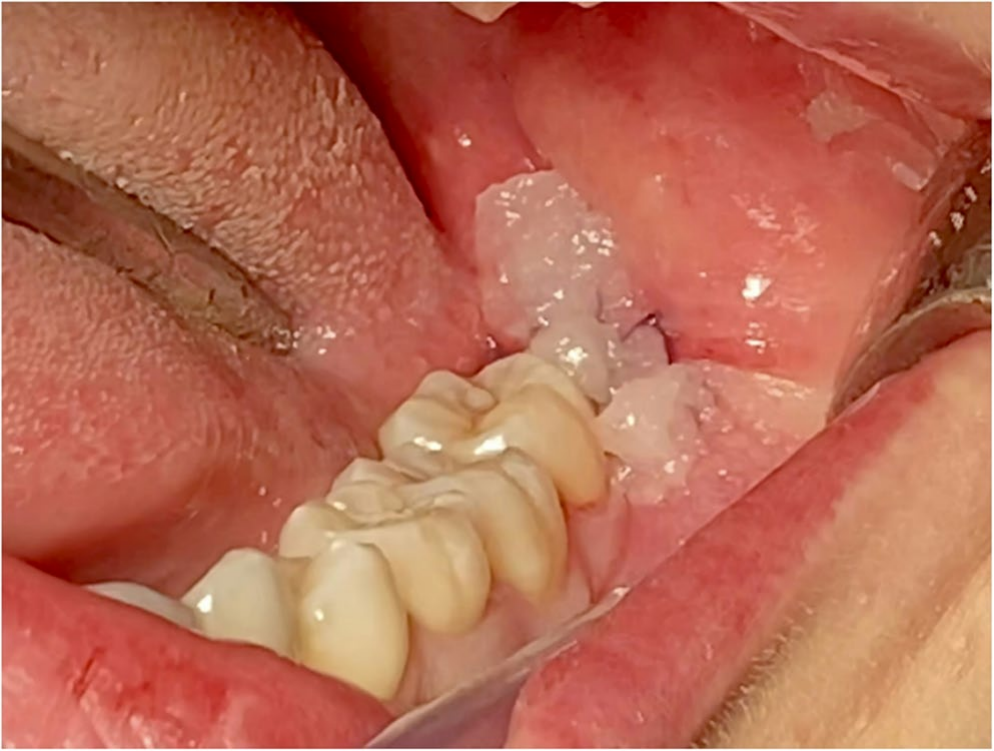
Application of topical ozone gel on the post-surgical wound

According to the randomization, at test sites, ozone in the gaseous state produced by a portable unit for oxygen ozone therapy (MEDICAL 99 IR^®^) was injected (concentration 5 µg/ml) after anesthesia around the incision (Fig. 2), in the post-extraction socket (40 µg/ml) after suturing (Figs. 3 and 4). In addition, patients were instructed on how to apply the ozone gel on the post-surgical site 2x day for 7 days after surgery starting the day after surgery. All patients received pre-operative antibiotic prophylaxis (2 tablets of amoxicillin + clavulanic acid 875 + 125 mg or clarithromycin 250 mg; 1 h before surgery) and continued the therapy after surgery (1 tablet; 2x day for 6 days). Analgesic therapy encompassed the used of ibuprofen tablets 600 mg every 8 h after meals the day of surgery and thereafter whenever needed. No corticosteroids were prescribed.

## Outcome measures

Evaluation of all parameters was performed after 1, 3, and 7 days after surgery by one examiner (P.B.) blinded to the treatment provided and not involved within the surgical procedure. The following outcomes were recorded.

Primary outcome: perceived pain, recorded at 1, 3, and 7 days after surgery using a Numerical Rating Scale (NRS), a written determination of a pain level on a scale from 0 to 10, having 0 as “no pain” and 10 as “excruciating pain” [25].

Secondary outcomes:

- Functional limitation in mouth opening (trisma) at 7 days after surgery measuring on day 0 (before the intervention) and on day 7 as the distance (in mm) between the upper central incisor and lower central incisor with a dedicated caliper.
- Number of analgesic tablets taken within the 7 days following surgery, recorded daily by the patient on a dedicated sheet.
- Patient’s perceived quality of life within the 7 days following the intervention, assessed with the Oral Health Impact Profile (OHIP-14) questionnaire completed by the patient on post-operative day 7 [8].

## Statistical analysis

Sample size calculation was performed assuming a power of 90% and a two-sided significance level of 5% to detect a clinically relevant difference in post-operative pain 7 days after surgery recorded using a Numerical Rating Scale (NRS), corresponding to a Cohen’s d of 0.5 using a paired-sample test and set within-subject correlation of 0.5.

This effect size was selected based on a previous study [15] which reported a larger effect. However, a more conservative estimate (d = 0.5) was adopted for the present calculation. The study sample was described with median and interquartile range for continuous variables and with absolute frequency and percentage for categorical variables. Differences between the treatment arms for the primary outcome measure and for the number of taken analgesics were carried out using the Wilcoxon signed rank test.

Secondary outcomes evaluated differences between the two treatments using a mixed-effects linear regression model taking into account the repeated measurement on the same patient. Primary and secondary outcomes were presented with their 95% confidence of interval. A significance level of 5% was considered a statistically significant and R software version R 4.0.5 was used for data analyses. The statistical analysis was performed by one of the authors (A.Ri) not involved in any part of the clinical investigation and blinded to the provided treatment.

## Results

### Subject accountability and participants characteristics

Of the originally 54 enrolled patients, 50 (20 males (40%) and 30 females (60%); median age: 21(19.00–23.00 years; 100% non-smokers) completed the study since 4 declined to participated to the study. The median surgical time was 27 (22–34) minutes for the test group and 25 (21–35) minutes for the control group. This difference was not statistically significant different (*p* = 0.21). Study flow-chart is displayed in Fig. 1.

### Primary and secondary outcome measures

**Fig. 5:**
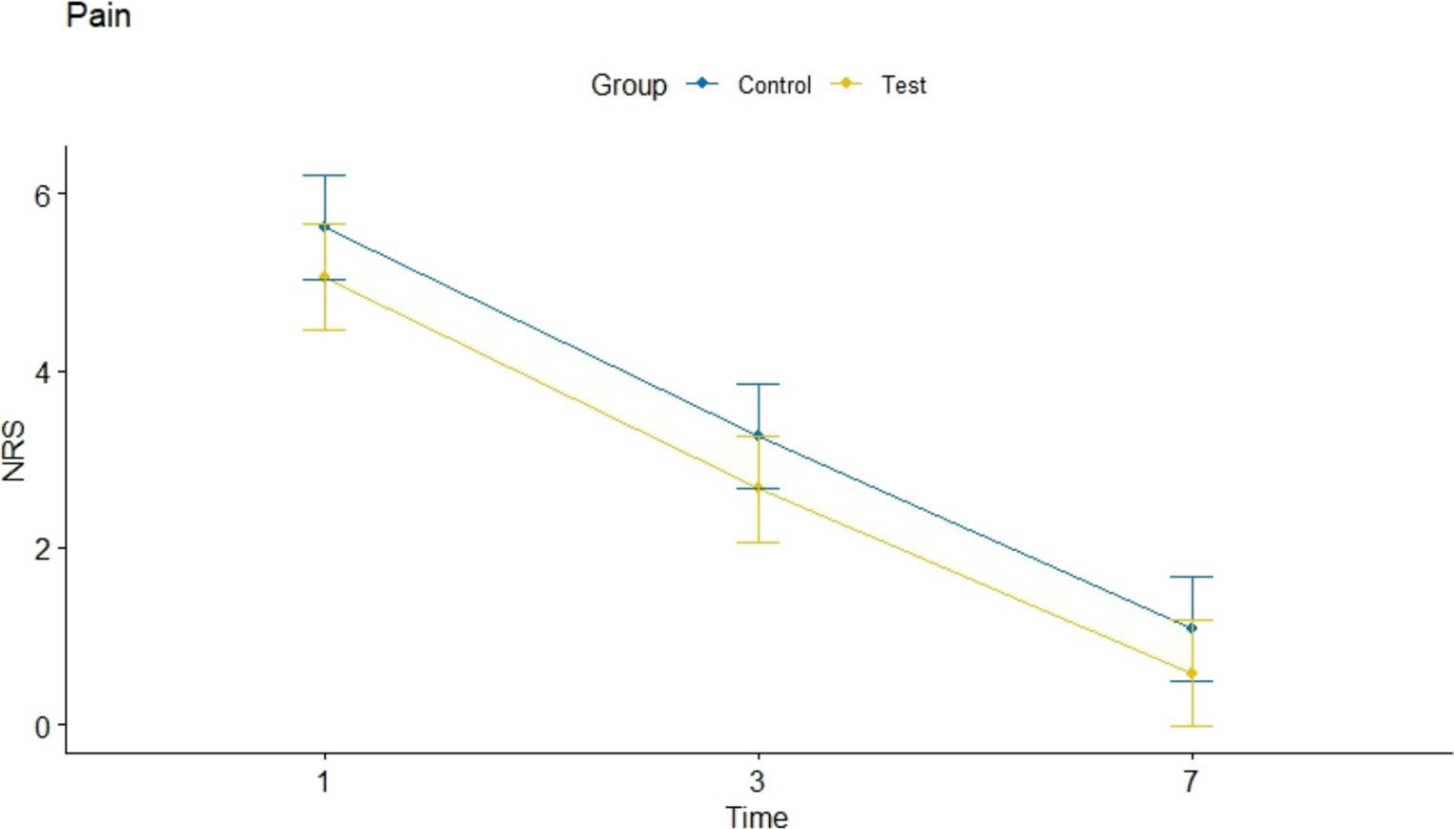
Median perceived pain through the 7-days within the two groups (Control (**A**); Test (**B**))

Patients’ perceived pain was in the control group 5.62 (95% CI:5.02–6.22) (day 1), 3.26 (95% CI:2.66–3.869 (day 3) and 1.08 (95% CI:0.48–1.68) (Day 7), while it was 5.06 (95% CI:4.46–5.66), 2.66 (95% CI:2.06–3.26) and 0.58 (95% CI:0–1.18.18) in the test group (Table 1). The test group reported a clinically relevant but not statistically significant decrease in post-operative pain scores on days 1 and 3 and non-significant at day 7. The difference between test and control was 0.56 (*p* = 0.09; 95% CI:0.00–1.21.00.21), 0.60 (*p* = 0.07; 95% CI:0.00–1.25.00.25) and 0.50 (*p* = 0.13; 95% CI: 0.00–1.15.00.15), respectively. No statistically significant differences were detected between the two groups at all time points (*p* > 0.05) (Table 1; Fig. 5).

**Table 1:** Median perceived pain at 1,3 and 7 days after surgery using a numerical rating scale (NRS) within the two groups

	Day 1	Day 3	Day 7
Control Group	5.62 [IC95%:5.02–6.22]	3.26 [IC95%:2.66–3.86]	1.08 [IC95%:0.48–1.68]
Test Group	5.06 [IC95%:4.46–5.66]	2.66 [IC95%:2.06–3.26]	0.58 [IC95%:0–1.18.18]
Difference	0.56 [IC95%:0.00–1.21.00.21]	0.60 [IC95%:0.00–1.25.00.25]	0.50 [IC95%:0.00–1.15.00.15)
P-value	> 0.05	> 0.05	> 0.05

*NRS* numeric pain rating scale

**Fig. 6:**
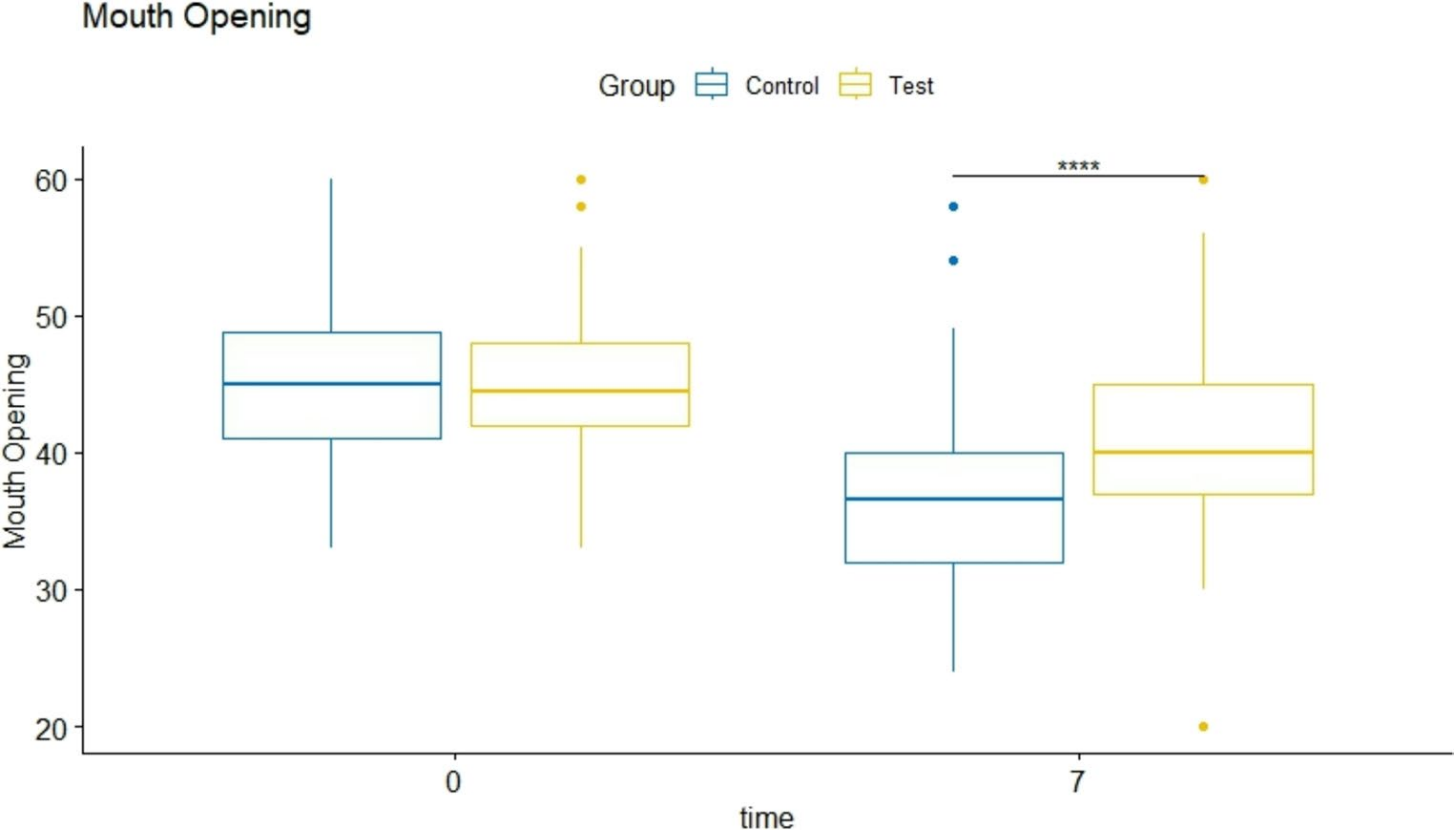
Mean mouth opening before surgery and at the 7-days visit within the control (**A**) and test (**B**) group

Initial mean mouth opening was 46 ± 6 mm in the test and 45 ± 6 mm in the control group, without any statistically significant difference (*p* = 0.8). At 7 days, the test groups displayed a value of 40.8 mm (95% CI:39.2–42.4) vs. 36.6 mm (95% CI:34.9–38.2) in the control group: this difference was statistically significant different in favor of the test group of 4.24 mm (*p* < 0.001; 95% CI: 6.55–1.93) compared to the control group (Fig. 6).

**Fig. 7:**
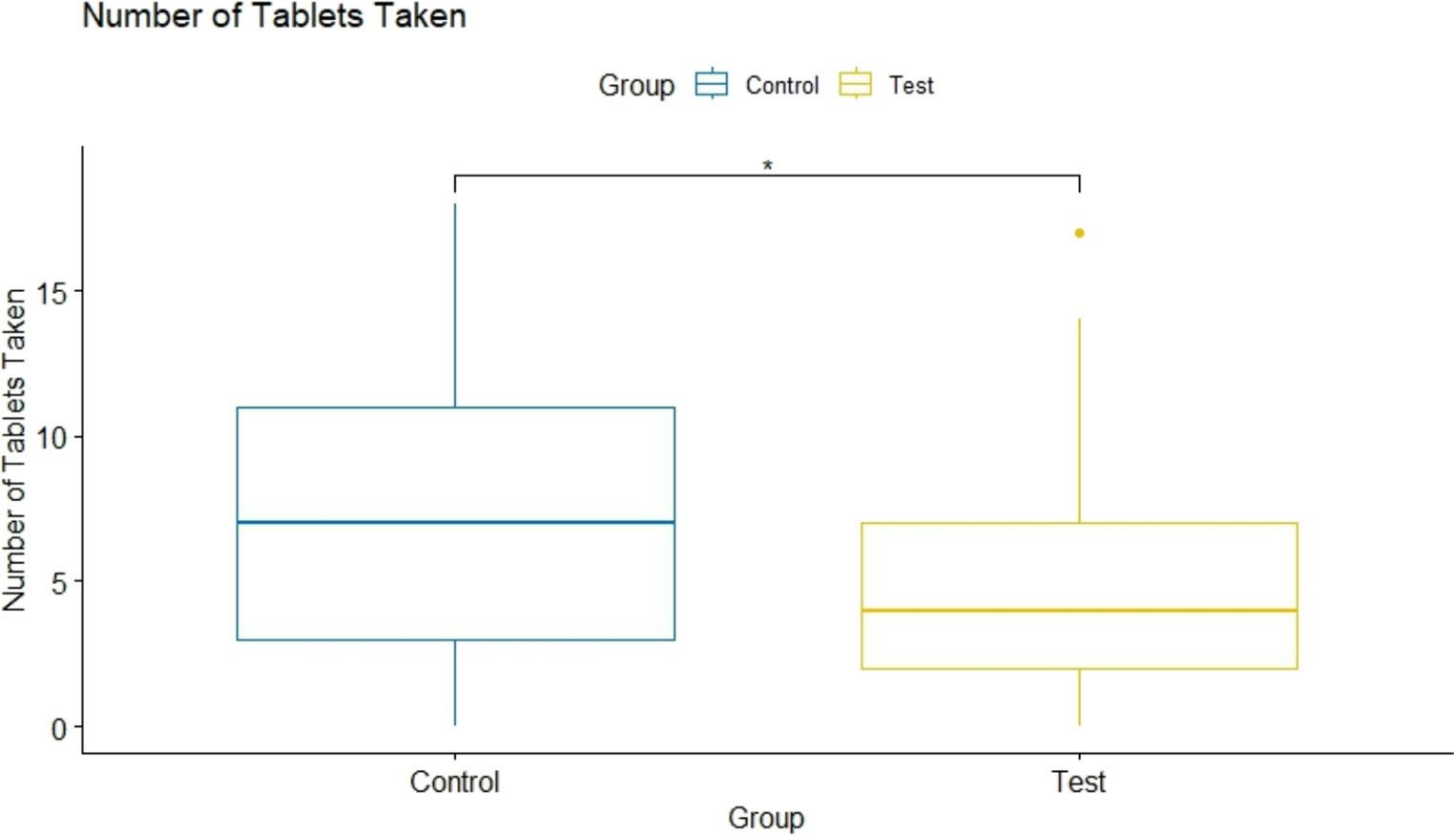
Number of tablets intake at 7-days post-op within the control (**A**) and test (**B**) group

**Fig. 8:**
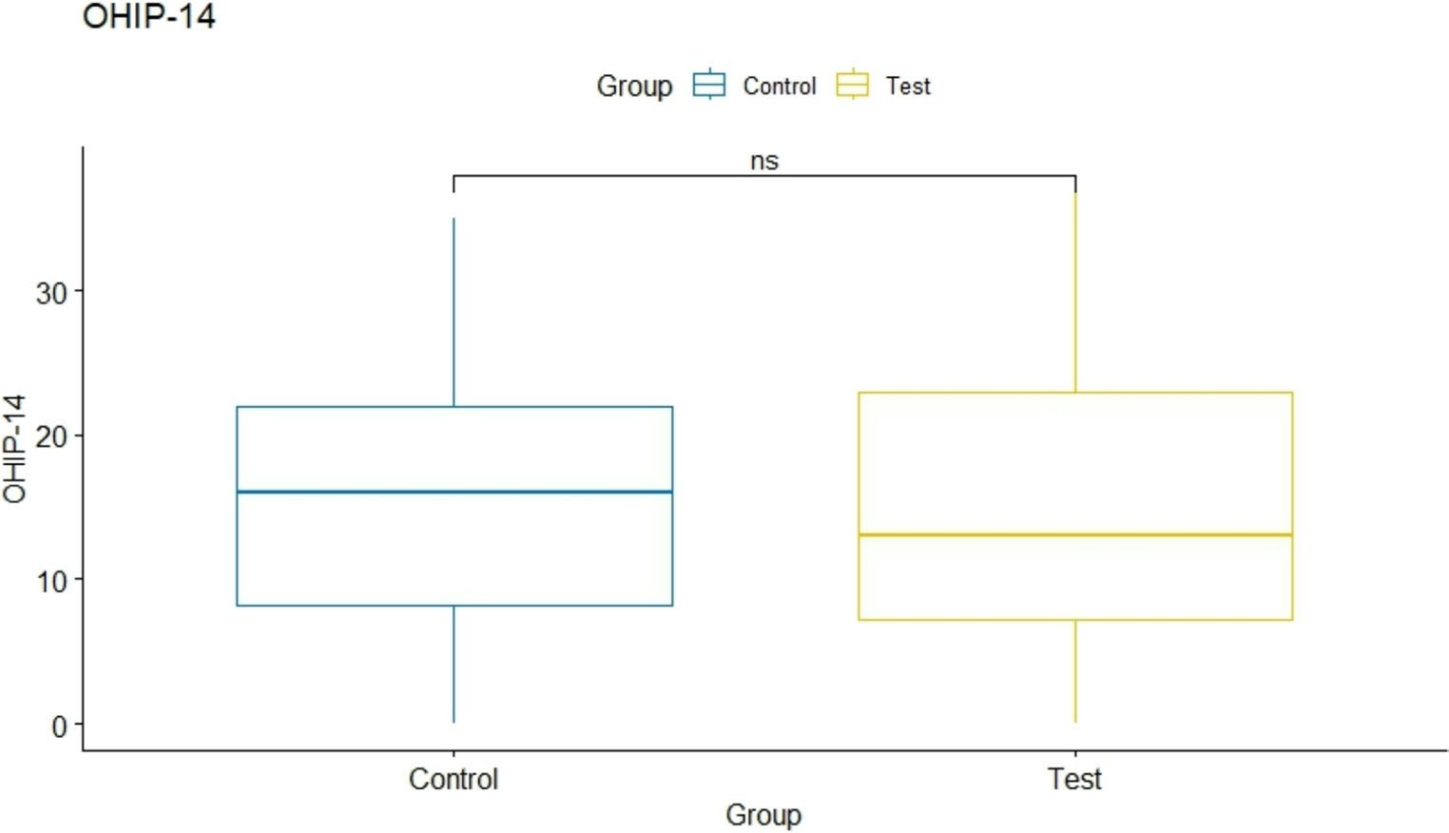
Mean OHIP-14 scores for the control (**A**) and test (**B**) group at the 7-days examination

At the 7 days post-op, the median number of analgesic tablets intake was 4 (95% IC: 3–6) within the test and 7 (95% IC: 5–9) in the control group. This difference was statistically significant (*p* = 0.01) (Fig. 7). Finally, the evaluation of the OHIP-14 questionnaire at day 7 post-operative, the mean score of the test was 13 (95% IC: 10–17) and 16 (95% IC: 11–20) in the control group, with no statistically significant difference between groups (*p* = 0.424) (Fig. 8).

## Discussion

The objective of this study was to evaluate the effectiveness of ozone therapy on post-operative pain, analyzing simultaneously the potential benefits on trisma, medication intake and quality of life. Based on the obtained results, it can be concluded that ozone therapy was proved to have beneficial clinical effects in terms of mouth opening and in number of analgesic tablets at 7 days after surgical removal of impacted mandibular third molar, despite the lack of adjunctive benefit on patients’ perceived pain reduction. The implemented method to measure the perceived pain (i.e., NRS) has been widely used, however, given the reported high heterogeneity in the assessment methods, definitive conclusion on this point cannot be drawn. Several factors have been correlated to post-operative sequelae such as surgery duration [20] and operator [15]: in the present study, test and control surgical time did not statistically differ, consequently removing this important potential bias from the interpretation of the obtained results. Moreover, all interventions were performed by two experienced oral surgeons, applying the same surgical protocol, with consequent minimal influence of operator’s effect on the recorded outcomes [15].

The present protocol does present elements of novelty such as the use of ozone in two forms: (a) the gaseous phase to administer higher concentrations of ozone at intervention, ensuring an immediate effect, followed by (b) a topical application, by means of ozonated gel, to ensure a prolonged effect on the surgical wound. The scientific rationale of such delivery was based on the evidence that molecules are higher stabile when in contact with the oral mucosa at body temperature [23]. One important finding is that no adverse events were recorded in the test group of ozone, neither during the surgical procedure, using ozone in gaseous form, nor in the period of home application of the gel, confirming previous evidence suggesting its safety and non-cytotoxicity on keratinocytes and fibroblasts [4, 14]. Nevertheless, few patients reported the gel taste as unpleasant of the gel.

With respect to the number of tablets taken, our results support the use of ozone therapy: as indicated by Naik et al., ozone is involved in the synthesis of biologically active substances such as interleukins, leukotrienes and prostaglandins, which are useful in reducing inflammation and pain [18]. In addition, the protective layer of ozone gel above the surgical site during the initial post-operative phase could prevent wound contamination, especially if used in conjunction with chlorhexidine [4], as in our protocol, and considerably reduce pain compared to the control side. One of the major post-operative complication after wisdom tooth removal is the development of trisma: our findings are confirmatory of previous data (Sivalingam, Panneerselvam, Raja, & Gopi [27]),, reporting a beneficial effect on the maximum mouth opening which allows better wound healing, facilitated by the anti-hypoxic, biosynthetic and analgesic properties of ozone [16, 29].

Limited mouth opening is attributable to the presence of edema, muscle fiber lesions, multiple needle penetrations, elevation and manipulation of flaps and presence of anesthetic or clot in muscle fibers, and usually peaks on the second day after surgery and resolves at the end of the first week (Balakrishnan, Narendar, Kavin, Venkataraman, & Gokulanathan [1]),. To evaluate it, we have adopted the OHIP-14 questionnaire, the quality of life perceived by patients in the 7 days following the intervention. We did not get any significant results, unlike Kazancioglu et al., comparing the study group with that control, most likely due to a great heterogeneity in the questionnaire responses [15]. Consequently, additional studies with larger sample are to be performed to detect potential differences.

Despite the split-mouth randomized design, this study does present some major limitations which have to be disclosed: first, this protocol included test sites operated first, did not include a specific assessment of post-operative swelling and the use of two ozone formulations (i.e., gaseous and oily) did not allow to discriminate which of the two could have had a greater impact on the evaluated outcomes. In addition, the lack of use of a placebo gel should be acknowledged as well as the potential limited effect of the prescribed therapy which relied on patient’s strict adhesion to the post-operative domiciliary regime. Finally, despite a priori power analysis, the lack of statistically significant difference with respect to the primary outcome measure might be advocated to the relatively small sample size.

## Conclusion

Ozone therapy was proved to have beneficial clinical effects in terms of mouth opening and in number of analgesic tablets at 7 days after surgical removal of impacted mandibular third molar, despite the lack of adjunctive benefit on patients’ perceived pain reduction. Consequently, its use as adjunct to surgical removal of impacted mandibular third molars might be considered following the presented promising results.

## Data Availability

The data that support the findings of this study are available upon reasonable request from the corresponding author. The data are not publicly available due to privacy or ethical restrictions.
